# Effectiveness of gold nanoparticles in prevention and treatment of oral mucositis in animal models: a systematic review

**DOI:** 10.1186/s13643-023-02425-9

**Published:** 2024-01-25

**Authors:** Minati Choudhury, Paul Brunton, Donald Schwass, Daniel Pletzer, Jithendra Ratnayake, George Dias, Geoffrey Tompkins

**Affiliations:** 1 Sir John Walsh Research Institute, Faculty of Dentistry, University of Otago, Dunedin, New Zealand; 2 Present Address: Restorative Dentistry, School of Dentistry, International Medical University, Kuala Lumpur, Malaysia; 3 Present Address: DVCA, Curtin University, Perth, Australia; 4 Department of Oral Rehabilitation, Faculty of Dentistry, University of Otago, Dunedin, New Zealand; 5 Department of Microbiology and Immunology, University of Otago, Dunedin, New Zealand; 6 Department of Oral Sciences, Faculty of Dentistry, University of Otago, Dunedin, New Zealand; 7 Department of Anatomy, University of Otago, Dunedin, New Zealand

**Keywords:** Gold nanoparticles, Oral mucositis, Animal model, Systematic review

## Abstract

**Background:**

Oral mucositis remains a significant complication during cancer therapy with no effective treatment. Gold nanoparticles offer anti-inflammatory, antioxidant properties with low toxicity. This study systematically reviews the literature assessing gold nanoparticles in the management of oral mucositis in animal models.

**Methods:**

A literature search was undertaken using MEDLINE, Embase, PubMed, and Web of Science databases, using the format for Systematic Review Centre for Laboratory Animal Experimentation. Prior to the review, the protocol was registered in the systematic review register, PROSPERO (registration no. CRD42021272169). Outcome measures included ulceration, histopathological scores, inflammatory mediators, microbial growth, and pain. Study quality was analysed by SYRCLE risk-of-bias tool.

**Results:**

Only one study met the inclusion criteria, documenting reduction in ulceration, inflammatory, and oxidative biomarkers. Exposure to AuNPs prevented inflammatory response induced by 5-fluorouracil in oral mucosa of hamsters. However, a high risk of bias necessitates further research.

**Conclusion:**

This review identifies a potential therapeutic strategy for prevention and management of oral mucositis. It also provides future direction for gold nanoparticle research in oral mucositis; however, there is lack of sufficient evidence to derive any conclusion. Research with standardized parameters including nanoparticle size, capping agent, surface charge, and appropriate oral mucositis animal models will establish risk–benefit balance and margin of safety for therapeutic use of gold nanoparticles for oral mucositis.

## Background

Oral mucositis (OM), the inflammation and ulceration of oral mucosa, affects up to 80% of patients undergoing head and neck cancer therapy [1, 2]. Mucosal ulcerations compromise nutrition and interrupt dosing in cancer therapy, increasing secondary infection risk and mortality, requiring parenteral nutrition, and lengthening the time and expense of hospital stays [1, 3]. OM evolves through a dynamic, pan-mucosal event [4, 5]. Radiation therapy (RT) and chemotherapy (CT) initiate DNA damage and release reactive oxygen species (ROS) in the underlying tissue. ROS activate and amplify key inflammatory mediators including nuclear factor-kappa B (NF-κB) which in turn induce pro-inflammatory cytokines, ultimately resulting in basal cell death and loss of mucosal integrity leading to bacterial colonization and sepsis [2, 4]. NF-κB, an innate immune transcription factor associated with inflammation, cell proliferation and apoptosis, plays a crucial role in OM pathogenesis [6, 7]. In the inactive state, NF-κB is retained in the cytoplasm by the inhibitor kappa B (IκB) proteins [6]. Ionizing radiation, chemotherapy, bacteria, and bacterial cell envelope products cause phosphorylation of IκB proteins by IκB kinase complex allowing nuclear translocation of NF-κB and subsequent gene upregulation leading to pro-inflammatory cytokine production (e.g. IL-1β, IL-6, and TNF-α) resulting in tissue injury [7]. These cytokines further activate NF-κB and increase inflammation [8, 9]. NF-κB also activates cyclooxygenase II (COX-2), an inducible enzyme that amplifies OM severity through prostaglandins that mediate pain and inflammation [10, 11]. There is a lack of consensus on the contribution of microorganisms to the initiation of OM [4]; however, microbial imbalance due to RT and CT may activate the innate immune system through NF-κB signalling, promoting an inflammatory cascade that ultimately leads to cell death and exacerbation of OM [12, 13]. Additionally, bacterial products penetrate the connective tissue and stimulate additional pro-inflammatory cytokines, exacerbating inflammation [14]. Despite the clinical significance, there is no effective way to either prevent or treat mucositis [15]. Current treatment strategies, including anti-inflammatory agents such as topical benzydamine HCL, cryotherapy, low-level laser therapy, and patient-controlled analgesics (morphine), are not consistently effective [15–18]. Hence, the development of an effective therapeutic strategy is a high priority.

ROS, NF-κB signalling, and pro-inflammatory cytokines (TNF-α, IL-1β, IL-6) play crucial roles in OM pathogenesis and progression. Thus, mechanistic strategies for OM prevention and management target these cellular pathways with anti-inflammatory and ROS-scavenging agents for therapeutic benefit [19–21]. Gold nanoparticles (AuNPs) offer unique size- and surface chemistry-dependent anti-inflammatory and antioxidant effects with low toxicity both in vitro and in vivo [11, 22, 23]. Their therapeutic effects are mostly attributed to inhibition of key transcription factors (NF-κB, COX-2) and subsequent reduction of pro-inflammatory cytokines (TNF-α, IL-1β, IL-6) [11]. AuNPs block nuclear translocation of NF-κB and cytokine induction by interacting with IκB kinase complex [24]. Additionally, AuNPs increase the expression of anti-inflammatory cytokine IL-10 [25]. Furthermore, ROS-scavenging nature of AuNPs through Kelch-like ECH-associated protein 1 (KEAP-1) and nuclear factor erythroid-2-related factor-2 (Nrf2) pathways are established in various animal models [26, 27]. Nrf2 transcription factor regulates expression of antioxidant enzymes such as glutathione (GSH), superoxide dismutase, and heme oxygenase 1 (HO-1) associated with reduced severity of OM [28]. In inactive conditions, Nrf2 binds to KEAP1 and degrades in the cytoplasm. AuNPs attenuate expression of *KEAP1*, stimulating nuclear translocation of Nrf2 with subsequent increase in gene expression of antioxidant enzymes that prevents and reduces ROS-generated inflammatory response [29]. In addition, GSH and HO-1 stimulated by AuNPs result in inhibition of NF-κB and consequent decreased inflammation. Thus, AuNPs may reduce the upregulation of inflammatory and oxidative pathways and limit OM severity.

The physicochemical properties such as particles size, shape, and surface chemistry profoundly affect the biological properties of AuNPs. Particles less than 10nm diameter are highly reactive providing greater anti-inflammatory response than larger particles, but may be more toxic because their greater surface area-to-volume ratio results in variations in particle response and increased cellular internalization, biodistribution, and accumulation [22, 23, 30–32]. Additionally, functionalization with specific ligands/capping agents alters the surface chemistry of AuNPs, influencing the cellular interaction and biologic properties [22, 33, 34]. Negatively charged AuNPs are essentially non-toxic and anti-inflammatory, whereas a positive surface charge not only invests antimicrobial activity but also makes the particles more toxic [35]. Thus, modulation of AuNPs size and surface chemistry could optimize them as therapeutic agent [22, 23, 30–32], but a lack of standardization of experimental design makes it difficult to derive definitive conclusions.

Systematic reviews aim to recover all information and identify knowledge gaps critical for subsequent clinical translation while eliminating any form of bias [15, 36–39]. Though not yet common practice, systematic review of experimental animal studies is essential to obtaining information on safety and efficacy of an intervention aimed at human disease [40]. Discrepancies between experimental data from animal studies and subsequent clinical trials have raised concerns regarding the validity of experiments with animals [41]. A systematic review provides transparency to the quality of published research and the translational value of preclinical studies. Therefore, the main objective of this study is to systematically review published accounts of the effects of AuNPs on the severity of chemotherapy- and/or radiation-induced oral mucositis in animal models. Animals with chemotherapy- and/or radiation therapy-induced oral mucositis receiving gold nanoparticles as the intervention were identified and compared to control groups (no treatment, placebo, vehicle treatment).

### Outcome measures

OM is characterized by painful oral ulcerations, inflammation, and microbial dysbiosis. Therefore, multiple outcome measures were evaluated to assess therapeutic efficacy of AuNPs. The primary objective was to evaluate the effect of AuNPs on the mucosal ulcerative lesions. However, subjective assessment of lesions makes a study comparison difficult. Hence, standardized scoring systems that objectively and reproducibly record OM severity were used. In addition, histopathologic grading of OM was also included to validate AuNP efficacy. Understanding the mode of action of AuNPs is important for developing therapeutic strategies. AuNPs inhibit key transcription factor NF-κB and inflammatory mediators COX2, TNF-α, IL-1β, IL-6, and IL-8 in vivo [11]. These inflammatory regulators were assessed as potential targets for OM prevention and management. Additionally, promotion of opportunistic pathogens exacerbates OM and may result in secondary infections [42, 43]. Smaller spherical AuNPs with positive surface charge are effective antimicrobials suggesting therapeutic benefit. Hence, effect of AuNPs on microbial growth was assessed in this review. Furthermore, OM is associated with severe pain that compromises nutritional intake leading to distress and decreased quality of life. Assessments of pain-related behaviours such as inactivity, eating and drinking, and social interaction can indicate efficacy of interventions. The effects of AuNPs on pain-related behavioural changes were also evaluated. Table 1 summarizes the outcome measures.

**Table 1:** Outcome measures studied

Number	Outcome measures
1	Changes in the size of ulcerative lesions
2	Histopathological changes of the oral mucosa
3	Reduction in the expression of inflammatory markers (TNFα, IL-1β, IL-6, IL-8)
4	Inhibition of key inflammatory pathways (NF-κB and COX2)
5	Reduction in microbial growth
6	Pain-related behavioural changes

## Methods

### Protocol development and registration

This systematic review was conducted following the Systematic Review Protocol for Animal Intervention Studies format issued by the Systematic Review Centre for Laboratory Animal Experimentation (SYRCLE) [44] and a validated and standardized protocol that defines the research question, search strategy, inclusion and exclusion criteria, data extraction format, and risk-of-bias assessment specifically for preclinical animal systematic reviews. It consists of 50 items in three sections. Section A summarizes general information such as title, authors, funding source, and potential conflict of interest. Section B pertains to the rationale and the research question. Section C is further divided into subsections formulating the review methodology as follows:

1. Identification of databases, search strategy, and animal search filters
2. Study selection based on inclusion and exclusion criteria
3. Data extraction consisting of study design, animal models, intervention characteristics, and outcome measures
4. Risk-of-bias assessment to determine study quality through tools such as SYRCLE’s risk-of-bias tool and CAMARADES’s study quality checklist
5. Data extraction
6. Data analysis/synthesis

The protocol was registered with PROSPERO, the international prospective register of systematic review protocols for clinical studies (registration no. CRD42021272169).

### Search strategy

A thorough and transparent step-by-step search strategy was adapted to identify all relevant animal studies involving gold nanoparticle intervention in oral mucositis [45]. The following electronic literature databases were searched using both Medical Subject Headings (MESH) terms and free-text terms: MEDLINE, Embase, PubMed, and Web of Science. Briefly, the search strategy was based on MeSH terms, text words, and word variants: “oral mucositis”, PubMed and EMBASE search filters for "animals”, “gold nanoparticles” and “anti-inflammatory agents”. The full search strategy is attached as a supplementary file. Grey literature was also searched through library databases and web searches for theses and conference presentations. There were neither language restrictions nor publication date restrictions. This minimized the reporting bias and imprecise study conclusions. Pre-screening was based on title/abstract followed by full-text screening. To prevent bias in the selection process, two reviewers screened each phase, and discrepancies were resolved by discussion. The searches were rechecked before the final analysis to retrieve studies eligible for inclusion.

### Inclusion and exclusion criteria

The inclusion and exclusion criteria were based on the PICO model (population, intervention, comparison and outcomes) and the study designs. Animal models (in vivo) including all species and sexes, with chemotherapy- and/or radiation therapy-induced oral mucositis receiving any type of AuNP as the intervention, were included. All timings, frequencies and dosages of AuNP were included. All studies with treatment before/after/in concurrence with oral mucositis model induction were considered. Animals with chemotherapy- and/or radiation therapy-induced oral mucositis receiving no treatment, a placebo, or a vehicle were the control population. Only original studies were considered. Animals with comorbidity, studies with no animal model or without an oral mucositis model, and ex vivo, in vitro, and in silico studies were excluded. Interventions that were not AuNPs and combination therapies were excluded. Studies with a control group receiving treatments other than vehicle, placebo, or no treatment were also excluded. Articles published in all languages and years were eligible.

### Study selection and data extraction

Screening of studies involved two phases: initial screening by title and abstract, followed by full-text screening. In each phase, two researchers independently assessed the article. Disagreement over the eligibility of particular studies was resolved through discussion. Data were entered on a template for assessment of study quality and evidence synthesis. Extracted information included study population, baseline characteristics, details of the intervention and control conditions, study methodology, outcomes and times of measurement, and suggested mechanisms of action. Meta-analysis was not appropriate as only one study qualified for final inclusion.

### Assessment of the risk of bias

Study quality was assessed by using SYRCLE’s risk-of-bias tool that evaluates whether the following information were reported and methods adequately described:

1. Randomization to treatment and control groups, i.e. did the investigation, involves a random sequence generation process
2. Sample size calculation
3. Appropriate investigator blinding
4. Reporting animal exclusions
5. Pre-determined inclusion/exclusion criteria

## Results

**Fig. 1:**
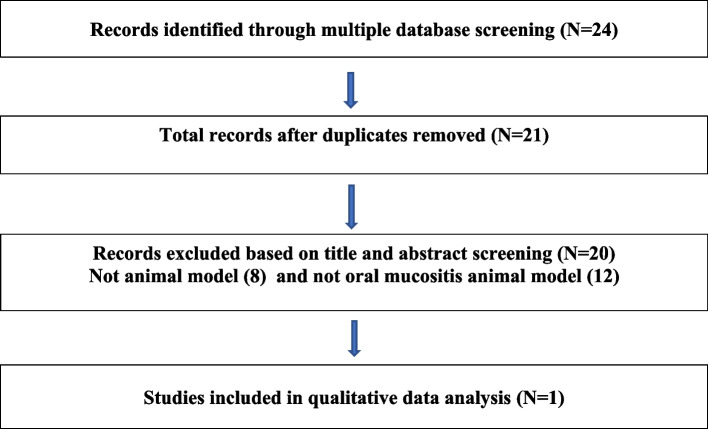
Flowchart of study selection

Of the 21 studies initially identified, only one qualified for inclusion (Fig. 1). The included study evaluated the effect of AuNP on OM induced by 5-flourouracil (5-FU) using a golden Syrian hamster model [29]. Appropriate animal ethical approval was granted prior to the start of the trial. AuNP (10 nm) stabilized by polyvinylpyrrolidone (PVP) were synthesized and adjusted to 100 μg/mL. The animals were divided into four groups with five animals in each group: (i) Control group without OM receiving PVP, (ii) without OM receiving PVP and mechanical trauma (MT), (iii) 5-FU- and MT-induced OM treated with PVP (30 min before OM induction) for 10 days, and (iv) 5-FU- and MT-induced OM treated with AuNP (62.5, 125, and 250 μg/kg body weight) 30 min before OM induction, for 10 days. 5-FU was delivered intraperitonially on days 1 and 2 to induce OM followed by mechanical trauma on the right cheek pouch mucosa on day 4 by superficial scratching using a 22-gauge needle tip. Animals were euthanized on day 10 for macroscopic and histopathological examination; quantification of pro-inflammatory cytokines and GSH [46, 47], immunohistochemical measurement of NF-κB and COX-2 expression, Western blot quantification of transforming growth factor β (TGF-β 1/2) and SMAD 2/3, gene expression by real-time polymerase chain reaction (qRT-PCR) for *KEAP1* and antioxidant enzymes (hemeoxygenase 1(*HMOX-1*), NAD (P) H quinone dehydrogenase 1 (*NQO1*)) and organ quantification of AuNP by inductive-coupled plasma optical emission spectrometry (ICP-OES).

PVA-capped AuNPs prevented and improved the clinical, histopathological, and biochemical markers of OM induced by 5-FU and MT in hamsters. As expected, the PVP group showed a healthy mucosa without either erosion or vasodilatation (Score 0) with a normal epithelium (Score 1), while MT caused mild erythema (Score 1) without erosion and discrete cellular infiltration (Score 1). OM was confirmed in the 5-FU group with cumulative ulceration extending to more than 50% of the mucosa (Score 4.5). Histopathology revealed increased inflammatory response with severe vasodilatation and inflammatory infiltration (Score 4). Treatment with AuNP resulted in decreased clinical and histological scores in hamsters with induced OM. Thus, mild vasodilatation and superficial erosion of mucosa were evident without ulceration (Score 2), with mild inflammatory infiltration and signs of re-epithelization (Score 1). Doses below 250μg AuNP/kg did not improve the OM scores compared to the control group. At 250 μg/kg, AuNPs inhibited the key inflammatory mediators involved in OM (NF-κB, COX-2, TNF-α, and IL-1β) indicating therapeutic potential. Exposure to AuNP also decreased TGF-β and SMAD 2/3 proteins. TGF-β exerts pro-inflammatory effect with delayed healing and disruption of cell proliferation through NF-κB and SMAD 2/3 signalling pathways. Hence, reduction of TGF-β and SMAD 2/3 expression indicate decreased inflammatory response. Furthermore, AuNP treatment resulted in ROS-scavenging activity, decreasing expression of *KEAP1* that releases Nrf2 factor, and subsequent increase of antioxidant enzymes. AuNP also increased GSH levels and expression of antioxidant enzymes *HMOX-1* and *NQO1*. ICP-OES revealed low levels of nanoparticle accumulation in the liver and kidney without pathological changes. In summary, AuNPs (250 μg/kg) prevented and reduced 5-FU-induced OM in hamsters without toxicity suggesting therapeutic application in OM.

## Discussion

Several strategies to treat OM have been instigated over the past two decades [48–53], but an effective treatment has yet to be established, and the search for new therapeutics continues [15]. AuNPs display anti-inflammatory and antioxidant effects in vitro and in vivo by reduction of pro-inflammatory cytokines promising therapeutic application in OM treatment [11, 25, 54, 55]. Additionally, AuNPs exhibit biofilm inhibitory activity against microorganisms relevant to OM [56–58]. Thus, evaluation of published research and identification of knowledge gaps is justified for subsequent clinical translation. This systematic review assesses the efficacy of AuNPs to either prevent or manage cancer therapy-induced OM in animal models.

Relevant database search recovered only one peer-reviewed report assessing AuNPs to treat OM in an animal model [29]. Delivered at 250 μg/kg body weight, PVP-stabilized AuNP (10 nm) effectively reduced 5-FU-induced OM severity in hamsters. The therapeutic efficacy is attributable to inhibition of key inflammatory mediators including NF-κB, COX-2, TGF-β, and SMAD 2/3 with subsequent reduction in pro-inflammatory cytokines TNF-α and IL-1β. Exposure to PVP-AuNP also upregulated the Nrf2 pathway and increased antioxidant enzymes (GSH, HO-1, and NADPH) indicating a ROS-scavenging potential that could be therapeutically beneficial. Nevertheless, toxicity is a concern for biomedical application, and published reports are conflicting with respect to AuNP toxicity, but small particle size and variations in surface chemistry can modulate therapeutic efficacy and toxicity [30, 59, 60]. Smaller AuNPs have greater surface area-to-volume ratio making them more reactive, enhancing anti-inflammatory effects but also increasing cytotoxicity [23]. On the other hand, capping agents with neutral or negative surface charge make AuNPs less toxic [61], while positive charged agent increases cell death [35]. Smaller AuNPs with modulation of surface chemistry could increase therapeutic efficacy while reducing toxicity. Thus, non-toxic PVP-capped AuNPs of 10 nm diameter are promising therapeutic agent for clinical translation for OM. Although there are no further studies directly assessing AuNPs in OM animal models to substantiate this conclusion, animal models of rheumatoid arthritis establish the anti-inflammatory and antioxidant properties [11, 25, 55].

Just as importantly, this review identifies knowledge gaps to be addressed in future OM research [62]. The identified study establishes AuNPs inhibit inflammatory and oxidative response in chemotherapy-induced OM, but not against either radiation-induced mucositis or OM induced by chemoradiotherapy. AuNPs have been used to enhance the effect of radiation treatment in cancer therapy [63], and it is prudent to evaluate toxicity and anti-inflammatory effects during radiation therapy and combination therapy in an animal model. Furthermore, microbial dysbiosis has emerged as an influential inflammatory mediator in OM and requires further assessment. Currently, there are no reports assessing the effect of AuNPs on the microbiota in an OM animal model. Furthermore, the study also highlights the importance of optimization of AuNPs for therapeutic benefit. The single identified study used appropriate controls and methodology as described, however, information regarding randomization, blinding, and sample size was not provided and therefore has a risk of bias. In fact, most animal studies lack essential details such as sample size calculation, randomization, and blinding [40]. In animal models, the pathology is induced; hence, timing of randomization is important to assess whether the disease was initiated before randomization or was randomly assigned to avoid selection bias [64]. Randomization of housing animals is also important because caging conditions (lighting, humidity, temperature) can influence outcomes. Randomization is also important to blind the animal care givers and investigators to avoid performance bias [64]. Furthermore, circadian rhythms influence biochemical parameters in animals and can affect the outcomes; hence, random selection for outcome assessment is essential regardless of the allocation of the test and control groups [64]. Additionally, correct sample size calculation must be undertaken and reported. A study involving few animals can miss the real effect of an intervention, whereas a large number of animals can lead to unnecessary wasting of the resources and consequent ethical concerns [65]. Without these details, results must be interpreted cautiously as it may under- or over-estimate the effect of the intervention. To this end, the PREPARE (Planning Research and Experimental Procedures and Animals: Recommendations for Excellence) and ARRIVE (Animal Research Reporting of In Vivo Experiments) guidelines should be followed to ensure study quality [66, 67].

## Conclusion and future directions

This review identifies AuNPs as a potential treatment strategy for prevention and management of OM. However, there is lack of research to derive a definitive conclusion. As AuNPs particle size, capping agents, and surface charge profoundly influence biologic properties, well-designed studies with standardized physicochemical parameters of AuNPs in appropriate animal models, including radiotherapy and chemoradiotherapy, are required to clarify the toxicity and therapeutic potential of AuNPs in prevention and management of OM. Recent studies focus on enhanced anti-inflammatory and antioxidant effects of smaller AuNPs (< 10 nm). Their higher surface area-to-volume ratio increases cellular interactions, internalization and biodistribution providing increased therapeutic efficacy at lower dose. Thus, smaller AuNPs targeting key inflammatory pathways such as NF-κB and Nrf2 have promising therapeutic effects for OM. Hence, the potential of ultrasmall AuNPs as a therapeutic agent for OM demands further investigation.

## Data Availability

The datasets during and/or analysed during the current study available from the corresponding author on reasonable request.
